# 2236. Effectiveness of TBP-PI-HBr in Patients with Instrumentation, Anatomic or Functional Abnormalities: Secondary Analysis from ADAPT-PO

**DOI:** 10.1093/ofid/ofac492.1854

**Published:** 2022-12-15

**Authors:** Angela K Talley, Paul B Eckburg, Ian A Critchley, Ian A Critchley, Gary E Moore, Nivedita Bhatt, Lori A Muir, Lori A Muir, David Melnick

**Affiliations:** Spero Therapeutics, Cambridge, Massachusetts; Spero Therapeutics, Cambridge, Massachusetts; Spero Therapeutics, Cambridge, Massachusetts; Spero Therapeutics, Cambridge, Massachusetts; Moore Computing Services, Inc., Little Rock, Arkansas; Spero Therapeutics, Cambridge, Massachusetts; Spero Therapeutics, Cambridge, Massachusetts; Spero Therapeutics, Cambridge, Massachusetts; Spero Therapeutics, Cambridge, Massachusetts

## Abstract

**Background:**

Tebipenem pivoxil hydrobromide (TBP-PI-HBr) is an oral carbapenem with activity against Enterobacterales uropathogens, including drug resistant strains. The ADAPT-PO trial demonstrated the non-inferiority of oral TBP-PI-HBr vs. intravenous (IV) ertapenem (ERT) in treating patients with cUTI and/or acute pyelonephritis (AP). Patients with certain anatomical disorders, functional (metabolic or neurological) disorders and/or urinary tract instrumentation (i.e., indwelling catheters) are at increased risk for poor outcomes and recurrent bacteriuria. This secondary analysis of ADAPT-PO evaluated outcomes in patients with various cUTI risk factors.

**Methods:**

ADAPT-PO was a Phase 3 multinational, double-blind, double-dummy trial evaluating oral TBP-PI-HBr vs. IV ERT in 1372 hospitalized adult patients with cUTI/AP. Patients were randomized 1:1 to oral TBP-PI-HBr or IV ERT for 7-10 days. The primary efficacy endpoint was overall response (composite favorable clinical and microbiologic response) in the microbiological intent-to-treat (micro-ITT) population. Overall outcomes were assessed among patients with identified with at least one cUTI risk factor at baseline.

**Results:**

At baseline, 382/449 (85.1%) and 361/449(86.2%) of patients in the TBP-PI-HBr and ERT arms, respectively had ≥1 risk factor and 289 (64.4%) and 268 (64.2%) had ≥2 risk factors. Anatomical abnormalities and/or urinary track instrumentation were present in 252 (56.1%) TBP-PI-HBr and 242 (57.8%) ERT patients; functional abnormalities were present in 90 (20.0%) and 85(20.3%) respectively). Among patients with ≥1 risk factor, overall response at TOC was 56.3% and 59.6% with TBP-PI-HBr and ERT, respectively, vs. 73.1% and 74.1% among those with no risk factors. Clinical response rates at TOC were high and similar in both arms in patients with (≥92.9%) and without (≥91.4%) risk factors whereas microbiologic responses were more variable between the 2 subsets.

**Conclusion:**

In cUTI patients with ≥1 risk factor for complicated disease at baseline, outcomes were comparable between oral TBP-PI-HBr and IV ERT. As expected, these patients were at higher risk for recurrent bacteriuria. Overall responses in both groups were higher among patients without these risk factors.

**Disclosures:**

**Angela K. Talley, MD**, Spero Therapeutics: Employee|Spero Therapeutics: Stocks/Bonds **Paul B. Eckburg, MD**, AN2 Therapeutics: Stocks/Bonds|Spero Therapeutics: Advisor/Consultant **Ian A. Critchley, PhD**, Spero Therapeutics: Employee|Spero Therapeutics: Stocks/Bonds **Ian A. Critchley, PhD**, Spero Therapeutics: Employee|Spero Therapeutics: Stocks/Bonds **Gary E. Moore, PhD**, Spero Therapeutics: Advisor/Consultant **Nivedita Bhatt, PhD**, Spero Therapeutics: Employee|Spero Therapeutics: Stocks/Bonds **Lori A. Muir, B.Sc.**, Spero Therapeutics: Employee|Spero Therapeutics: Stocks/Bonds **Lori A. Muir, B.Sc.**, Spero Therapeutics: Employee|Spero Therapeutics: Stocks/Bonds **David Melnick, MD**, Spero Therapeutics: Employee|Spero Therapeutics: Stocks/Bonds.

